# Cut-off point for high dysphonia risk in children based on the Child Dysphonia Risk Screening Protocol: preliminary findings

**DOI:** 10.6061/clinics/2020/e1682

**Published:** 2020-08-21

**Authors:** Giovana Kaila Santos Batista, Marcia Simões-Zenari, Kátia Nemr

**Affiliations:** Departamento de Fisioterapia, Fonoaudiologia e Terapia Ocupacional, Faculdade de Medicina FMUSP, Universidade de Sao Paulo, Sao Paulo, SP, BR.

**Keywords:** Voice, Voice Disorders, Child, Vocal Quality, Acoustic Analysis

## Abstract

**OBJECTIVES:**

The objective of the study was to establish a cut-off point for high dysphonia risk in children using the Child Dysphonia Risk Screening Protocol (DRSP-C).

**METHODS:**

Through a preliminary study, voice recordings of 59 children (4-12 years of age) were collected during an auditory-perceptual analysis using the Consensus Auditory-Perceptual Evaluation of Voice protocol. Thirty of the patients had voice disorders (patient group), and 29 did not (control group). A risk score for dysphonia was then calculated, and data were compared between groups. The relationship between overall degrees of deviation and questionnaire scores was analysed. The questionnaire’s validity was verified from the area under the Receiver Operating Characteristic (ROC) curve, and cut-off points were obtained based on diagnostic criteria for screening procedures.

**RESULTS:**

The DRSP-C score was found to be higher for the patient group, as was the partial score for vocal behaviour. No correlation was found between overall degrees of dysphonia and questionnaire scores. The area under the ROC curve was measured as 0.678, denoting limited diagnostic capacity. The cut-off point was set at 16.50. Thus, above this value, dysphonia risk is higher.

**CONCLUSION:**

A cut-off point for high dysphonia risk was calculated. The DRSP-C proved to be a promising tool for children’s clinical vocal and health promotion and should be used in conjunction with General Dysphonia Risk Screening.

## INTRODUCTION

The anatomy and histology of the paediatric larynx are not fixed. Airway enlargement and changes in collagen distribution and vocal fold size denote that the paediatric larynx is evolving until the maturation of neurological control contributes to voice stabilization (1). Children tend to exhibit intense vocal behaviour, which may result in dysphonia (2).

The occurrence of dysphonia in the paediatric population ranges from 4.4 to 38% and depends on age, the definition of dysphonia and assessment protocols (3). The prevalence of dysphonia in children is higher in boys aged 8 to 14 years (3). In children aged 4 to 12 years, this range runs from 6 to 23% (4,5,6) with behavioural dysphonia being more prevalent due to an excessive and inappropriate use of voice. Phonotrauma is often observed in children and may result in the formation of lesions such as nodules on and edema of the vocal folds (5,6,7). The presence of congenital abnormalities may also affect voice quality, especially when associated with phonotrauma. Vocal fold cysts are the most common congenital disorder affecting this age group (8,9).

Voice emission is influenced by personality and environmental and social factors, such as the place that a child occupies in the family and how the voice is heard (4), which may also contribute to the occurrence of behavioural dysphonia. Other potentially risky factors to be highlighted include auditory alterations, gastroesophageal reflux, and respiratory diseases (7) in addition to allergic rhinitis and persistent cough, which may favour an increase in negative vocal symptoms (10).

The presence of dysphonia can negatively impact the quality of life of children, interfering with their social, affective and emotional well-being. It can create school and functional disadvantages that can extend into adulthood (4,5,6,11). Impacts of negative judgements of their voices by peers and adults can also affect their emotional well-being (4,12). Children and adolescents perceive a greater difference between their voices and those of their peers and often receive comments about their voices (4).

In the vocal assessment of children, studies have been dedicated to analysing the impact of voice disorders on children's well-being using questionnaires such as the Paediatric Voice-Related Quality of Life (pVRQOL) survey (13). The Glottal Function Index questionnaire also allows one to detect the presence of vocal alteration (3).

Child vocal assessment should employ an understanding of risk factors and their possible associations with the presence of dysphonia, guiding specialists in relation to best practices and guidelines. It is necessary to consider specificities of child behaviour and voice production based on the use of specific protocols for this age group (14), which remain limited (12).

In the literature consulted, no instrument that could calculate dysphonia risk in children was found. The Child Dysphonia Risk Screening Protocol (DRSP-C) was developed and tested in a pilot study and differentiated children with and without voice disorders, and its applicability to this population was proven (15). As it is a complementary questionnaire, its use must be in association with the General Dysphonia Risk Screening Protocol (DRSP-G) (16). The combined use of both questionnaires enables the investigation of general and specific factors related to vocal production.

The DRSP-G includes general questions related to the history of vocal alterations, comorbidities, vocal signs and symptoms, drug use, and contact with smokers, among others. It contains 18 questions that can be answered by individuals of any age, gender, level of education and use of voice. For score calculation, each response score ranges from 0 to 3 with 0 representing a positive response and scores of 1 to 3 representing negative responses ranging from least (1) to most (3) negative. With a visual analogue scale, a value measured using a millimetre ruler is added to the overall score. Each set of questions generates a partial score from the simple sum, and the sum of all scores yields the total score. Final scores range from 0 to 131 where the higher the score, the greater the risk. The survey has a high diagnostic capacity even when applied to children for whom a sensitivity value of 0.955 and a specificity value of 0.947 are observed. The cut-off point for high dysphonia risk in children is set at 22.50. Thus, it is not necessary for the score to be high for dysphonia risk to be high (16).

Robust instruments that can differentiate individuals with and without dysphonia and especially those aimed at children are valuable to vocal clinics due a lack of tests of this population. In addition, establishing cut-off points for high dysphonia risk allows for the use of this type of questionnaire for health promotion actions as a first phase of investigation. They are also of use to vocal clinics as a therapeutic follow-up measure.

The DRSP-C investigates specific aspects related to childhood such as vocal behaviour, playing with intense vocal use and the presence of other communication problems such as phonological and fluency disorders (15).

The objective of the present study was to establish a cut-off point for high dysphonia risk in children using the DRSP-C, to compare groups with and without vocal changes in relation to partial and total scores and to analyse the correlation between the degree of dysphonia and total scores.

## METHODS

This cross-sectional observational study was approved by the Institutional Research Ethics Committee (CAPPesp HCFMUSP 0560/10).

The project involved extensive research with three well-defined phases: the development of the instrument and an analysis of its applicability (15); the application of the questionnaire to part of the sample to test its differentiation between children with and without vocal alterations and to start modelling the cut-off point from the Receiver Operating Characteristic (ROC) curve; and questionnaire’s application to the full sample defined from sample calculations followed by speech and language and otorhinolaryngological assessments for a final analysis of the specified cut-off point.

From our sample calculations we found that to achieve 80% statistical power, at least 48 children needed to be included in each group. For the sample size used, the statistical power was calculated as 59.4%.

A total of 59 children participated in this phase of the study: 33 boys and 26 girls aged 4 to 12 years (mean of 8.1 years). Children with voice disorders had been evaluated at the Speech Therapy Voice Research Laboratory (LIF Voice) of the University of Sao Paulo’s Faculty of Medicine. Data for this evaluation were obtained from LIF Voz medical records. Children in the control group attended schools in the region to which the researchers had access.

All guardians authorized participation and signed an informed consent form. Terms of assent were used with the children. Exclusion criteria: diagnosis of other communication problems or comorbidities interfering with voice production such as syndromes, neurological disorders, ear infections, the flu, colds, respiratory problems, and gastroesophageal reflux.

Children with vocal disorders (GVA) had an otorhinolaryngological diagnosis and had undergone a complete vocal assessment at LIF Voice. For the perceptual-auditory classification of vocal quality, their voices were recorded using a unidirectional and condensed headset microphone (AKG 520, Germany) positioned approximately 3 cm away from the mouth and connected to a desktop computer with an Edirol UA-101 interface. The free Audacity^®^ software programme (https://audacityteam.org) was used for voice recording.

Children without vocal disorders (GNVA) had no complaints or history of dysphonia. To confirm the absence of changes, their voices were recorded at the school. An iPad (MP2F2BZ/A, iOS 10.3.3) with the Shure Motiv application (Shure, 44.100 Hz, monosound in WAV format) was used and connected to a microphone (Shure MOTIV MV88 unidirectional) positioned 3 cm from the child’s mouth.

Consensus Auditory-Perceptual Evaluation of Voice (CAPE-V) protocol tasks (17) translated into Brazilian Portuguese (18) were requested in both situations. All recordings were analysed by a speech therapist specializing in voice and with high reliability using this protocol (intraclass correlation coefficient of 0.975) (16). An overall level of dysphonia of 35.5 or more denoted the presence of vocal disorders (19).

For both recordings, the children were seated in a chair appropriate for their size so that they were seated upright with their backs resting on the backrest with their arms at their sides. All recordings were collected in an acoustically controlled environment using the SoundMeter noise measurement application developed by Digital SoundMeter while keeping noise levels at below 50 dB. A comparative test of recordings taken from both sets of equipment was conducted, and it was determined that the same level of quality was maintained.

Thirty children (50.9%) composed the GVA, of which 17 were female (56.7%) and 13 were male (43.3%); the GNVA included 29 children (49.1%), of which eight were female (31%) and 20 were male (69%).

Those responsible responded to the DRSP-C (15).

The DRSP-C (Appendix 1) includes specific questions about a child's vocal behaviour and aspects related to hearing, vocally more active personality characteristics, school and family routines that may interfere with vocal production, and other communication disorders. As in the DRSP-G, each response score ranges from 0 to 3 with 0 representing a positive response and scores of 1 to 3 representing negative responses ranging from least (1) to most (3) negative. The scores for each block of questions form partial scores. The final score, from the simple sum of all partial scores, varies from 0 to 45. Similar to the DRSP-G (16), the higher the score, the greater the risk. Both questionnaires provide quantitative and qualitative data. For the construction of the DRSP-C, steps enabling evidence of validity based on the content of the test and on relationships to other variables were considered (20) based on an extensive literature review and pilot study (15). At this stage, questions regarding diagnostic accuracy were analysed as recommended for the development of instruments of this nature (20).

For statistical analysis, SPSS Statistics software version 25.0 (IBM Corp., Armonk, NY, EUA) was used. For the calculation of 95% confidence intervals, the corrected and accelerated bias method based on a 2000-sample bootstrap was used. For sample calculations, G*Power software version 3.1.9.2, was used.

A student’s test for independent samples, Mann-Whitney U test and Fisher’s exact test were used to compare the groups in relation to partial and total scores defined from an analysis of adherence to normality performed via the Shapiro-Wilk test.

Pearson’s correlation tests were used to analyse the relationship between the overall degree of deviation and questionnaire scores. Values of *p*≤0.05 were considered significant.

To analyse the validity of the DRSP-C in relation to the perceptual-auditory analysis by the CAPE-V protocol, the area under the ROC curve was measured. The area under the ROC curve (AUC-ROC) measures a test’s performance in relation to the gold standard, i.e., the capacity for a new test to discriminate between sick and healthy individuals (21) and in this case between children with and without dysphonia. The higher the AUC-ROC and the closer it is to 1, the greater the capacity for the new test to discriminate between the occurrence of the event of interest. The closer the ROC curve is to the diagonal line, the worse the discriminatory power of the test (22).

Values related to scores obtained for the two groups and sensitivity, specificity, efficiency, false positive and negative, and positive and negative predictive values of the DRSP-C formed the basis for defining the cut-off point for high-risk dysphonia and were performed based on Weihing and Atcherson (23). Due to the instrument’s screening characteristics, sensitivity was prioritized over specificities of the cut-off point.

## RESULTS

Fifty-nine children aged 5 to 12 years (mean of 8.06 years) participated. In total, 33 were boys (aged 5 to 12, average of 7.8 years) and 26 were girls (aged 5 to 12, average of 8.4 years).

The GVA and GNVA groups were similar in age (averages of 7.67 and 8.48 years, respectively, *p*=0.134).

The groups differed in partial scores for vocal behaviour and DRSP-C scores (Table 1).

No correlation was found between degrees of vocal alteration and DRSP-C scores (Table 2).

**Figure 1:**
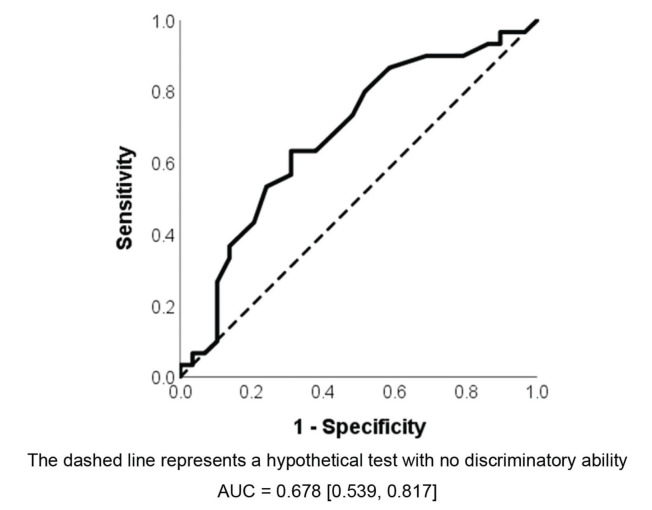
ROC curve for the overall DRSP-C score comparing children with and without voice disorders.

Regarding the test’s validity, the AUC-ROC statistic was measured as 0.678 [CI95% 0.539-0.817] (Figure 1), which is slightly above 0.50 but still far from 1. A child with vocal disorders was found to be 67.8% more likely to have a higher score on the DRSP-C compared to a child without vocal disorders.

As a cut-off point of 16.50 was obtained for the DRSP-C, any value higher than this point indicates a high degree of dysphonia risk. This point corresponds with a sensitivity value of 70.0%, a specificity value of 55.2%, a false positive of 44.8%, a false negative of 30.0%, an efficiency value of 62.6%, a positive predictive value of 61.8% and a negative predictive value of 64.0%.

## DISCUSSION

In the last decades, the field of speech therapy has evolved in its development of surveys to validate questionnaires mainly due to continuous studies in essential areas such as statistics and psychometry.

The present study sought to advance calls for a questionnaire that investigates dysphonia risk in children.

We must first highlight the composition of the sample used. The two groups studied were balanced in terms of gender and age, rendering them comparable. While we are still preliminary stages of research, we believe that the sample size used, while a limitation, made it possible for us to carry out necessary tests of the questionnaire’s accuracy values. Children’s participation in research is often hampered by poor adherence and availability among families and/or schools (24). Even when an agreement is reached, most children contacted do not fit inclusion criteria. Other research centres focused on children will be invited to apply the questionnaire in the next phase of the study.

The only partial score found to differentiate the two groups is vocal behaviour. Our data reinforce the influence of vocal behaviour on the development of dysphonia in children, corroborating the literature (5,6,7,8,25). Features such as speaking with effort, speaking without resting and imitating characters’ voices have previously been related to vocal alteration (26). On the other hand, speaking loudly (25,26) and shouting (26) can be observed in children regardless of the presence of vocal disorders. Therefore, it is important to analyse a set of vocal behaviours as done in the present study to develop a partial questionnaire score.

Other aspects measured by the questionnaire were not found to be as relevant to the occurrence of dysphonia as vocal behaviour, though they qualitatively measure important aspects and illustrate the multifactoriality of dysphonia by considering physical, emotional and environmental factors (26).

The DRSP-C score differentiated the groups and is configured as an important attribute of the instrument. The screening questionnaires, both general and specific, were designed to be complementary to auditory-perceptual, acoustic and physiological evaluations of voice (16) and to assist with relevant, quantitative and qualitative data and with indicators for clinical and health promoting actions. In addition, the DRSP-C should be applied in conjunction with the DRSP-G, and in a previous study of the DRSP-G for children the AUC-ROC was found to be very close to 1 (16), demonstrating its high degree of diagnostic accuracy (22). The importance of the DRSP-G and DRSP-C’s joint use is also reinforced by the fact that the latter, when applied alone, generates an AUC-ROC value more distant from 1, denoting an instrument with more limited diagnostic accuracy (22).

The present study also shows that it is not the number of aspects that causes a greater or lesser degree of dysphonia, i.e., children participating in this study did not show a relationship between questionnaire scores and the severity of vocal deviation. This result reinforces the need to explore these data qualitatively while considering what is most relevant to each child.

The cut-off point was set at 16.50, which, within the range of 0 to 45 that the questionnaire allows for, can be considered low. As in the DRSP-G, for this questionnaire it is not necessary for scores to be high for dysphonia risk to be high (16). Many negative aspects do not necessarily interfere with voice production, reinforcing the need for health promoting actions involving families, schools and children. In the same way that the presence of vocal alterations cannot be considered part of a child’s normal development, the presence of risk factors for dysphonia must also not be minimized (27). It is worth noting that this cut-off point was defined considering higher levels of sensitivity than specificity, denoting the survey’s greater capacity to detect children with vocal disorders though with a great chance of detecting false positives (21). Children identified above the cut-off point are to be referred for specific vocal assessment where cases can be confirmed, false positives can be identified and conduct can be defined.

The individual questionnaire cannot be considered a robust test (22) even though it exhibits a remarkable capacity to differentiate between altered and normal cases. It proves promising in the joint analysis of dysphonia risk when used with the DRSP-G and further research will analyse this joint application.

The proposed cut-off points can guide actions to promote health, enabling the characterization of dysphonia risk in children of different populations in addition to their use in clinical settings during evaluation and follow-up with children in speech therapy due to vocal problems. The use of specific protocols with children is necessary (14), and the findings of this study reinforce this conclusion.

Actions involving children and their families can be guided by these data and are recommended, as it is known that vocal alterations can interfere with voice-related quality of life (3) and can often be underestimated by parents (4).

Voice disorders restrict the communicative behaviours of children and their general well-being in addition to affecting their social and academic lives (4,5,6); therefore, it is important to develop new tools for the management of this disease in this population as proposed in this study.

Information generated by the two questionnaires, the DRSP-G and DRSP-C, may contribute to a more accurate assessment of intervention and reinforce the need to improve strategies for motivating behavioural changes in children's vocal therapy. Further work must confirm the study of these scores and apply the proposed cut-off point to a larger sample.

## CONCLUSION

A cut-off point for high dysphonia risk was calculated. The evaluated questionnaire was found to be efficient in identifying children with vocal disorders and partial scores related to vocal behaviour and associated with the presence of vocal disorders.

The DRSP-C serves as an interesting tool for children’s vocal clinic and health promotion actions and should be used in conjunction with the DRSP-G and be evaluated by voice for those at high risk of developing dysphonia.

## AUTHOR CONTRIBUTIONS

All authors contributed substantially to all of the following: (1) the study’s conception and design, data acquisition, or data analysis and interpretation; (2) manuscript drafting or revising for key intellectual content; and (3) final approval of the manuscript version submitted.

## Figures and Tables

**Table 1:** Descriptive values and comparative analysis according to values of variables of the DRSP-C

Variable	Group	n	Mean	SD	Median	Min.	Max.	*p*	ES
VB	GNVA	29	9.17 [7.38. 11.14]	5.39	8.00 [8.00. 8.00]	1.00	23.00	0.010*^b^	0.334^r^
	GVA	30	12.67 [10.60. 14.67]	5.77	13.50 [11.50. 15.00]	1.00	25.00		
HE	GNVA	29	1.76 [1.34. 2.17]	1.15	2.00 [2.00. 2.00]	0.00	4.00	0.780^b^	0.038^r^
	GVA	30	1.63 [1.30. 1.99]	1.07	2.00 [2.00. 2.00]	0.00	4.00		
PE	GNVA	29	3.00 [2.59. 3.45]	1.31	3.00 [2.00. 3.00]	1.00	6.00	0.969^b^	0.005^r^
	GVA	30	2.93 [2.567. 3.33]	1.20	3.00 [2.50. 3.00]	1.00	5.00		
DRSP-C score	GNVA	29	16.10 [13.81. 18.55]	6.55	15.00 [13.00. 18.00]	5.00	33.00	0.029*^a^	0.585^d^
	GVA	30	19.93 [17.63. 22.20]	6.61	21.00 [19.50. 21.50]	5.00	36.00		

Variable	Group	n	Median	Min.	Q1	Q3	Max.	*p*	ES
PP	GNVA	29	1.00 [1.00. 2.00]	0.00	1.00	2.00	2.00	0.334^b^	0.131
	GVA	30	1.50 [1.00. 2.00]	0.00	0.75	2.00	3.00		

Variable	Categories	Group						*p*	
		GNVA n (%)		GVA n (%)		Total n (%)			
CO	0	24 (82.76)		20 (66.67)		44 (74.58)		0.233^c^	
	1	5 (17.24)		10 (33.33)		15 (25.42)			
CD	0	8 (27.59)		10 (33.33)		18 (30.51)		0.779^c^	
	1	21 (72.41)		20 (66.67)		41 (69.49)			
RO	0	23 (79.31)		17 (56.67)		40 (67.80)		0.095^c^	
	1	6 (20.69)		13 (43.33)		19 (32.20)			

Student’s t test for independent samples (^a^), Mann-Whitney U test (^b^) and Fisher’s exact test (^c^), (^d^) effect size measured using the coefficient d, (^r^) effect size measured using the coefficient r.

Legend: SD: Standard deviation; Min.: Minimum; Max.: Maximum; Q1:Quartile 1; Q3: Quartile 3; *: Statistically significant at 5% (*p*≤0.05); ES: Effect size.

**Table 2:** Correlation analysis between DRSP-C score and the overall degree of the CAPE-V.

Var.		DRSP-C
Overall Degree CAPE-V	Coefficient	0.199 [-0.035. 0.445]
	*p*	0.131

Pearson’s correlation analysis.
